# X-ray-determined structure of the technetium com­plex [Tc_2_(μ-CO)_2_(NC_5_H_5_)_2_(CO)_6_] revisited: [Tc_2_(μ-OMe)_2_(NC_5_H_5_)_2_(CO)_6_] as the correct formulation

**DOI:** 10.1107/S2053229623007957

**Published:** 2023-09-18

**Authors:** Maaz Zuhayra, Arne Lützen, Miguel A. Ruiz

**Affiliations:** a Klinik für Nuklearmedizin, Universitätsklinikum Schleswig-Holstein, Campus Kiel, Arnold-Heller-Strasse 9, D-24105 Kiel, Germany; b Kekulé-Institut für Organische Chemie and Biochemie, Universität Bonn, Gerhard-Domagk-Strasse 1, D-53121 Bonn, Germany; c Departamento de Química Orgánica e Inorgánica/IUQOEM, Universidad de Oviedo, E-33071 Oviedo, Spain; Cinvestav, Mexico

**Keywords:** technetium, crystal structure, carbon­yl, pyridine, methoxide

## Abstract

Some of us reported previously the structure of di-μ-carbonyl-bis­[tricarbon­yl(pyridine)­technetium], [Tc_2_(μ-CO)_2_(C_5_H_5_N)_2_(CO)_6_], as the main product of the reaction of [Tc_2_(CO)_10_] with pyridine at room temperature, using the reagent itself as solvent [Zuhayra *et al.* (2008). *Inorg. Chem.* **47**, 10177–10182]. On the basis of an X-ray analysis of the product, a mol­ecular structure was proposed with two bridging carbonyls displaying very unusual geometrical features, not explained at the time. Subsequent chemical considerations, coupled with density functional theory (DFT) calculations, prompted us to revise the original structure determination. Using the original raw diffraction data, we have now per­form­ed new refinements to show that the previously proposed ‘bridging car­bon­yls’ actually correspond to bridging methoxide groups, and that the crystals analyzed at the time therefore would correspond to the com­plex di-μ-methoxido-bis­[tricarbon­yl(pyridine)­technetium], *syn*-[Tc_2_(μ-OMe)_2_(NC_5_H_5_)_2_(CO)_6_]. This methoxide-bridged com­plex likely was a minor side product formed along with the main product in the above reaction, perhaps due to the presence of trace amounts of methanol and air in the reaction mixture.

## Abstract 2

New processing of the raw diffraction data of a com­pound formulated in 2008 as [Tc_2_(μ-CO)_2_(NC_5_H_5_)_2_(CO)_6_] has revealed that the bridging ligands were actually methoxide. The formation of [Tc_2_(μ-OMe)_2_(NC_5_H_5_)_2_(CO)_6_] in the room-temperature reaction of [Tc_2_(CO)_10_] with pyridine might have been facilitated at the time by the presence of trace amounts of methanol and air in the reaction mixture.

## Introduction

**Figure 1:**
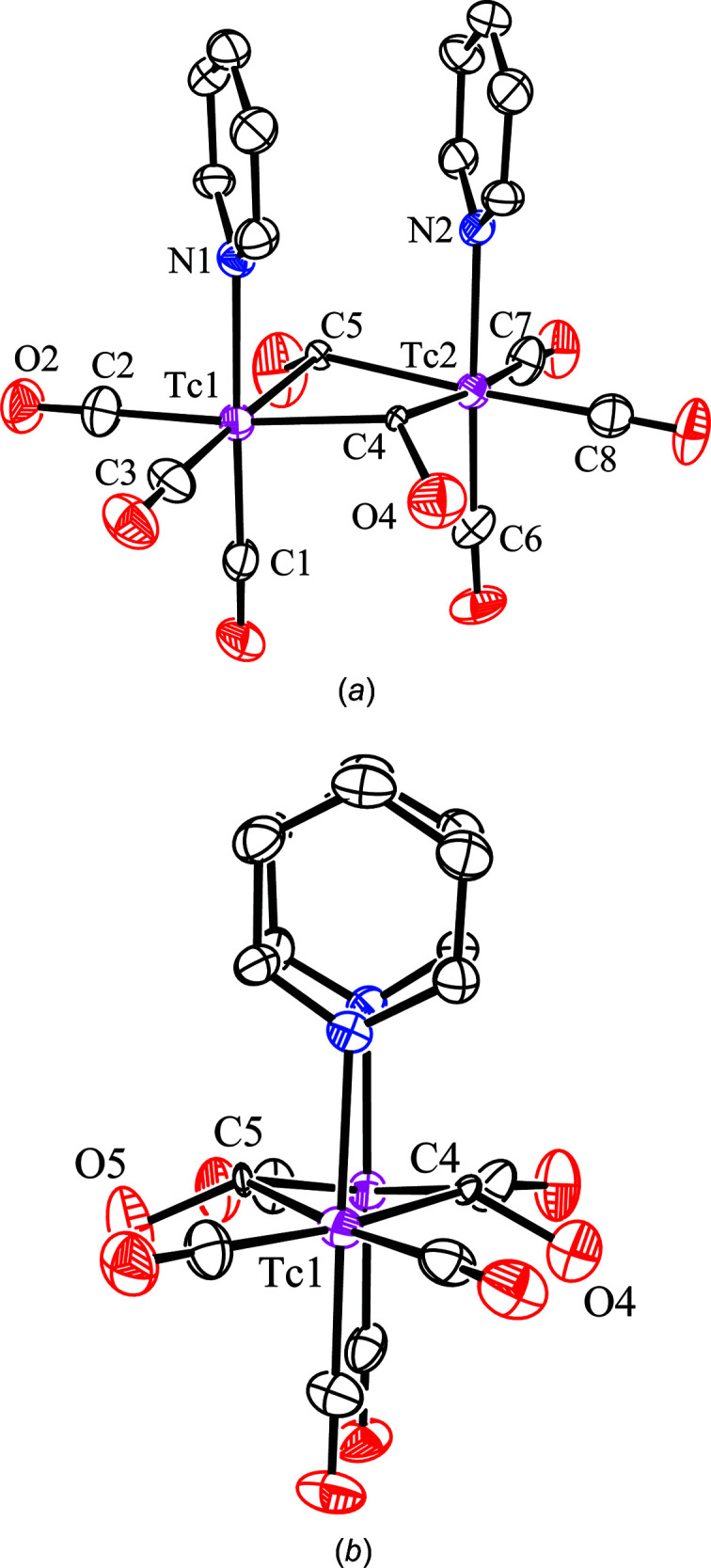
(*a*) The mol­ecular structure (30% probability displacement ellipsoids) of the presumed com­pound ***syn*** **-1**, with H atoms omitted for clarity. (*b*) A view of the mol­ecule along an axis close to the inter­metallic line (García-Vivó & Ruiz, 2020 ▸). Both images were generated from the original CIF file (Zuhayra *et al.*, 2008 ▸). Selected bond lengths (Å): Tc1⋯Tc2 = 3.370 (3), C1—O1 = 1.148 (13), C2—O2 = 1.14 (2), C3—O3 = 1.149 (15), C4—O4 = 1.451 (14) and C5—O5 = 1.470 (14).

Some of us reported previously that the room-temperature reaction of [Tc_2_(CO)_10_] with pyridine, using the reagent itself as solvent, yields the octa­carbonyl com­plex [Tc_2_(NC_5_H_5_)_2_(CO)_8_] (**1**) as the unique product, which upon heating undergoes an inter­esting C—H bond cleavage of a pyridine mol­ecule (Zuhayra *et al.*, 2008 ▸). On the basis of an X-ray analysis of the above product, a mol­ecular structure was pro­posed for isomer ***syn***-**1** with two bridging carbonyls dis­playing several unusual geometrical features not explained at the time (Fig. 1 ▸): (i) a strong pyramidalization of the bridgehead C atoms, with unusually small displacement parameters and very large C—O separations of *ca* 1.45 Å, actually close to the reference value of 1.42 Å for a C(*sp* ^3^)—O single bond (Cordero *et al.*, 2008 ▸), and far larger than the reference value of 1.21 Å for a double bond between these atoms (Pyykkö & Atsumi, 2009 ▸); and (ii) a large inter­metallic separation of *ca* 3.37 Å, far above that of the parent com­plex [Tc_2_(CO)_10_] (*ca* 3.03 Å; Bailey & Dahl, 1965 ▸; Sidorenko *et al.*, 2011 ▸), and inconsistent with the formulation of a single Tc—Tc bond, as required by application of the 18-electron rule to com­plex ***syn***-**1**. Recently, we used density functional theory (DFT) calculations to find that the most likely structure of **1** would display only terminal carbonyls and a staggered conformation, as observed in the parent precursor, and that the crystals anal­ysed by X-ray diffraction in 2008 would most likely cor­respond to either the hydro­peroxide-bridged ditechnetium(I) com­plex *syn*-[Tc_2_(μ-OOH)_2_(NC_5_H_5_)_2_(CO)_6_] (***syn*** **-2**) or its methoxide-bridged analogue *syn*-[Tc_2_(μ-OMe)_2_(NC_5_H_5_)_2_(CO)_6_] (***syn*** **-3**) (García-Vivó & Ruiz, 2020 ▸). This prompted us to revise the structure determination of com­pound ***syn*** **-1** by per­forming new refinements using the original raw diffraction data, which is the purpose of the present article. As will be shown below, the new refinements indicate beyond doubt that the crystal actually analyzed at the time was not that of com­pound ***syn*** **-1** but that of the methoxide-bridged com­plex *syn*-[Tc_2_(μ-OMe)_2_(NC_5_H_5_)_2_(CO)_6_] (***syn*** **-3**), whereby the ‘anoma­lous’ geometrical parameters mentioned above now become ‘as expected’.

## Experimental

Diffraction data were collected on a Siemens Nicolet Syntex R3m/V diffractometer using graphite-monochromated Mo *K*α radiation (λ = 0.71073 Å). Intensities were measured by fine-slicing φ-scans and corrected for background, polarization and Lorentz effects. The original structure of ***syn*** **-1** was solved by direct methods and refined with the programs *SHELXS86* and *SHELXL93* (Sheldrick, 2008 ▸) by a full-matrix least-squares method based on *F* ^2^ (Zuhayra *et al.*, 2008 ▸).

Taking the same diffraction data, the structures of ***syn*** **-2** and ***syn*** **-3** were now solved by a dual-space algorithm using *SHELXT2014* (Sheldrick, 2015*a* ▸) and refined by full-matrix least-squares on *F* ^2^ using *SHELXL2017* (Sheldrick, 2015*b* ▸) within *OLEX2* (Dolomanov *et al.*, 2009 ▸) and *WinGX* (Farrugia, 2012 ▸) environments.

### Refinement

Crystal data, data collection and structure refinement details for ***syn*** **-1**, ***syn*** **-2** and ***syn*** **-3** are summarized in Table 1 ▸. All carbon-bound H atoms were calculated at their optimal positions and treated as riding on their parent atoms using isotropic displacement parameters 1.2 (or 1.5 in the case of methyl groups) times larger than the *U*_eq_ values of the respective parent atoms. The methyl groups in ***syn*** **-3** were calculated as idealized rotating groups. We could not recover from the stored old data (recorded some 20 years ago) all the information currently required for standard CIF files, and this caused the appearance of some A-level alerts in the corresponding *checkCIF* reports for ***syn*** **-2** and ***syn*** **-3**.

## Results and discussion

**Figure 2:**
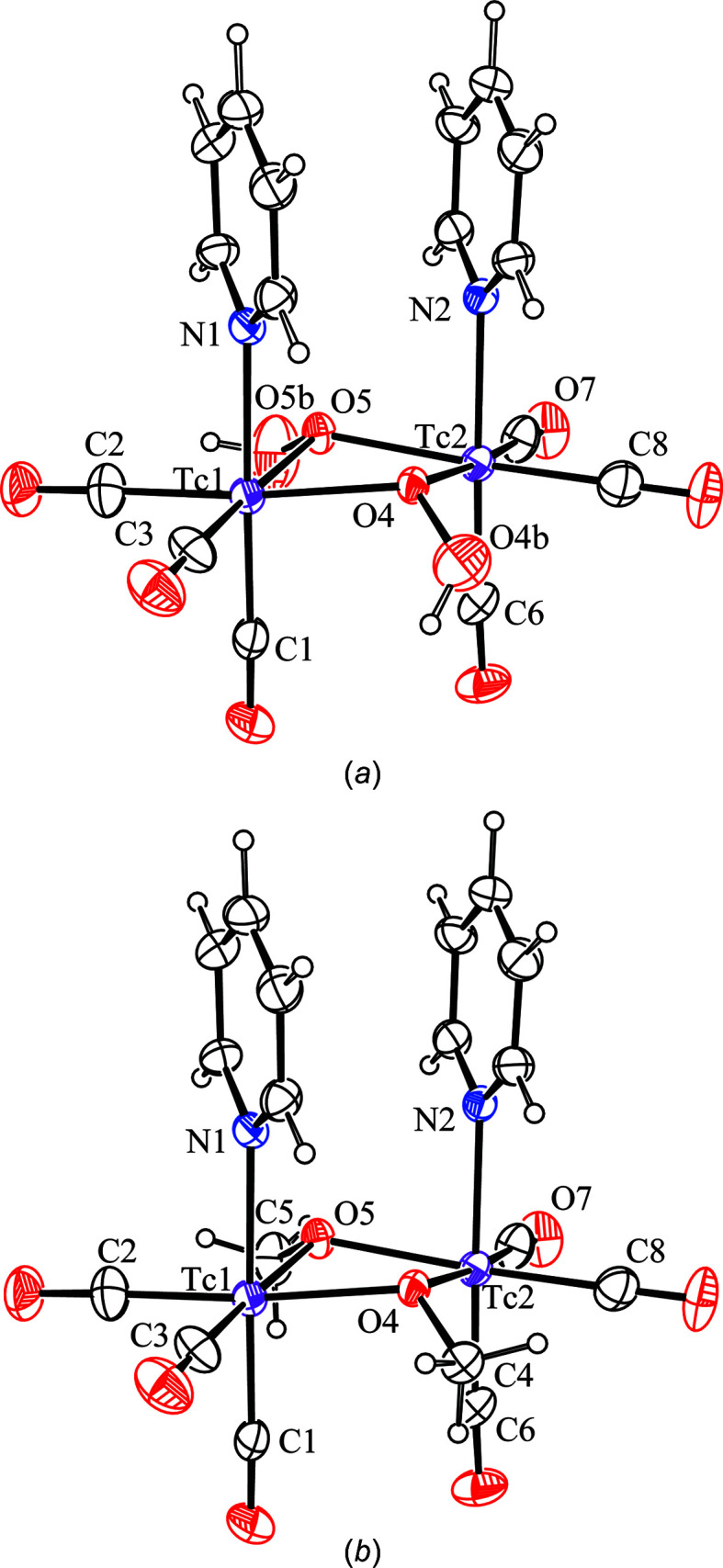
The mol­ecular structure (30% probability displacement ellipsoids) fol­lowing formulations as (*a*) ***syn*** **-2** and (*b*) ***syn*** **-3**.

The small size of the displacement ellipsoids of the bridgehead ‘carbon’ atoms (C4 and C5) in the original structure determination of ***syn*** **-1**, com­pared to those of the corresponding O atoms (O4 and O5; Fig. 1 ▸ and Table 2 ▸), suggested that positions C4 and C5 might actually correspond to atoms having a higher number of electrons (Stout & Jensen, 1989 ▸). Moreover, the theoretical calculations mentioned above indicated that replacing the bridging carbonyl ligands in ***syn*** **-1** with either OOH (peroxide) or OMe (methoxide) groups would yield com­plexes with geometries matching the anomalous features of the original structural determination (García-Vivó & Ruiz, 2020 ▸). We then proceeded to make new refinements with the original raw diffraction data under both hypotheses (***syn*** **-2** and ***syn*** **-3**, respectively). Both refinements converged satisfactorily to give improved fitting parameters, com­pared to the original refinement based on the formulation *syn*-[Tc_2_(μ-CO)_2_(NC_5_H_5_)_2_(CO)_6_] (Fig. 2 ▸, and Tables 1 ▸ and 2 ▸), but there were some significant differences between them: (i) the *R*_1_, *wR*_2_ and goodness-of-fit (GOF) values were better for ***syn*** **-3**. (ii) the *U*_eq_ values for the heavy atoms at the bridging positions (OO or OC) were more similar to each other in the case of ***syn*** **-3**; in contrast, the *U*_eq_ values for the O(H) atoms in ***syn*** **-2** were almost three times the value of the corresponding bridgehead O atom. This is clearly reflected in the significantly smaller values of *ca* 0.01 Å^2^ in the difference between the mean-square displacement amplitudes (ΔMSDA) for the C4/O4 or C5/O5 pairs in ***syn*** **-3**, as expected for mutually bonded atoms (Hirshfeld, 1976 ▸), which can be com­pared with values of *ca* 0.04 Å^2^ for the corresponding pairs in either ***syn*** **-2** or ***syn*** **-1** (Table 2 ▸). Moreover, we note that the average C—O bond length for the bridging methoxide groups in ***syn*** **-3** (*ca* 1.42 Å) exactly matches the reference value for a C(*sp* ^3^)—O single bond. In contrast, the average O—O bond length of 1.43 Å in the formulation as ***syn*** **-2** falls below the values of 1.45–1.50 Å typically determined for OO*R*-bridged com­plexes (García-Vivó & Ruiz, 2020 ▸). All of this provides conclusive evidence for the presence of methoxide groups bridging the Tc atoms in the com­plex under discussion. It is thus concluded that the crystal analyzed at the time actually was not one of com­pound ***syn*** **-1** but one of the methoxide-bridged com­plex *syn*-[Tc_2_(μ-OMe)_2_(NC_5_H_5_)_2_(CO)_6_] (***syn*** **-3**). We finally note that the geometrical parameters ob­tained for this com­plex are similar to those determined previously for different rhenium com­plexes with dimetal cores of the type *syn*-[Re_2_(μ-O*R*)_2_ *L*_2_(CO)_6_] having bridging alkoxide or hydroxide ligands and terminal pyridine, dipyridyl and poly­pyridyl ligands (García-Vivó & Ruiz, 2020 ▸). The latter belong to a relatively large family of com­plexes which have been studied extensively because of their photophysical and chemical properties, host–guest inter­actions and biological activity.

After concluding that the crystal analyzed at the time, formed through crystallization from acetone/*n*-hexane of the bulk product obtained when reacting [Tc_2_(CO)_10_] with pyridine at room temperature, corresponds to the methoxide-bridged com­plex ***syn*** **-3** rather than the simple substitution product ***syn*** **-1**, the question then to be answered is from where could the methoxide ligands possibly arise. Unfortunately, we are not in a position to reproduce the above synthetic pro­cedure in our laboratories, so we can only speculate about its possible origin. We currently trust that com­plex ***syn*** **-3** might just have been a very minor side product formed along with the major product, which just happened to crystallize first from the reaction mixture. Inter­estingly, we note that many dirhenium polypyridyl com­plexes with metal cores of the type *syn*-[Re_2_(μ-O*R*)_2_ *L*_2_(CO)_6_] have been made by reacting [Re_2_(CO)_10_] with stoichiometric amounts of the pertinent N-donor ligand in the corresponding alcohol (*R*OH) or water, although high temperatures are typically required to form these pro­ducts. However, a separate experiment carried out previously by us revealed that stirring [Re_2_(CO)_10_] in pyridine at room tem­perature for 4 d caused no detectable transformation on the Re_2_ substrate, unless air is admitted into the reaction flask (García-Vivó & Ruiz, 2020 ▸). Based on the above indirect pieces of evidence, we tend now to think that formation of the methoxide-bridged com­plex ***syn*** **-3** during the slow reaction of [Tc_2_(CO)_10_] with pyridine at room temperature (5 d) might have followed from the presence of trace amounts of methanol and air in the reaction mixture.

## Conclusion

The raw diffraction data of the com­pound formulated in 2008 as *syn*-[Tc_2_(μ-CO)_2_(NC_5_H_5_)_2_(CO)_6_] have now been re-pro­cessed under the hypothesis that the bridging ligands might actually be either hydro­peroxide or methoxide ligands. The latter option proved to be the correct one, as it leads not only to better agreement parameters, such as *R*, *wR* or GOF, but also to chemically more sensible inter­atomic distances and displacement parameters for the non-H atoms of the bridging ligands. The formation of *syn*-[Tc_2_(μ-OMe)_2_(NC_5_H_5_)_2_(CO)_6_] in the room-temperature reaction of [Tc_2_(CO)_10_] with pyridine might have been facilitated at the time by the presence of unnoticed trace amounts of methanol and air in the reaction mixture.

## Supplementary Material

Crystal structure: contains datablock(s) syn2, syn3, global. DOI: 10.1107/S2053229623007957/zo3037sup1.cif

Structure factors: contains datablock(s) syn2. DOI: 10.1107/S2053229623007957/zo3037syn2sup2.hkl

Structure factors: contains datablock(s) syn3. DOI: 10.1107/S2053229623007957/zo3037syn3sup3.hkl

CCDC references: 142167, 2294431, 2294833

## Figures and Tables

**Table 1:** Experimental details for structural determinations of com­plexes ***syn*-1** to ***syn*-3**

	****syn**** **-1** ^*a*^	****syn**** **-2**	****syn*-3***
Crystal data			
Chemical formula	C_18_H_10_N_2_O_8_Tc_2_	C_16_H_12_N_2_O_10_Tc_2_	C_18_H_16_N_2_O_8_Tc_2_
*M*_r_	578.28	588.28	584.33
Crystal system, space group	Ortho­rhom­bic, *Pna*2_1_	Ortho­rhom­bic, *Pna*2_1_	Ortho­rhom­bic, *Pna*2_1_
Temperature (K)	200	200	200
*a*, *b*, *c* (Å)	18.116 (16), 10.359 (8), 12.148 (10)	18.116 (16), 10.359 (8), 12.148 (10)	18.116 (16), 10.359 (8), 12.148 (10)
*V* (Å^3^)	2280 (3)	2280 (3)	2280 (3)
*Z*	4	4	4
Radiation type	Mo *K*α	Mo *K*α	Mo *K*α
μ (mm^−1^)	1.25	1.26	1.26
Crystal size (mm)	0.3 × 0.2 × 0.2	0.3 × 0.2 × 0.2	0.3 × 0.2 × 0.2
			
Data collection			
No. of measured, independent and observed [*I* > 2σ(*I*)] reflections	2614, 2614, 2265	2614, 2614, 2265	2614, 2614, 2265
(sin θ/λ)_max_ (Å^−1^)	0.639	0.639	0.639
			
Refinement			
*R*[*F* ^2^ > 2σ(*F* ^2^)], *wR*(*F* ^2^), *S*	0.052, 0.146, 1.10	0.046, 0.122, 1.05	0.042, 0.113, 1.05
No. of reflections	2614	2614	2614
No. of parameters	273	281	274
No. of restraints	1	4	1
H-atom treatment	Only H-atom displacement parameters refined	Only H-atom displacement parameters refined	H-atom parameters constrained
Δρ_max_, Δρ_min_ (e Å^−3^)	1.16, −1.14	1.12, −0.97	1.13, −1.03
Flack parameter	0.07 (10)	0.04 (9)	0.02 (8)

Note: (*a*) data taken from Zuhayra *et al.* (2008 ▸). Computer programs: *SHELXL2017* (Sheldrick, 2015*b* ▸) in *WinGX* (Farrugia, 2012 ▸), *SHELXT2014* (Sheldrick, 2015*a* ▸) and *OLEX2* (Dolomanov *et al.*, 2009 ▸).

**Table 2:** Selected parameters (Å, Å^2^) for structural determinations following formulations as ***syn*-1** to ***syn*-3**

Parameter	****syn**** **-1** *^*a*^*	****syn**** **-2**	****syn**** **-3**
	(*XY* = CO)	(*XY* = OO)	(*XY* = OC)
Tc⋯Tc	3.370 (3)	3.369 (3)	3.368 (3)
Average Tc—(μ-*X*)	2.163	2.162	2.163
*X*4—*Y*4	1.451 (14)	1.441 (12)	1.424 (11)
*X*5—*Y*5	1.470 (14)	1.433 (14)	1.415 (11)
*U*_eq_(*X*4)	0.018 (2)	0.034 (2)	0.035 (2)
*U*_eq_(*X*5)	0.019 (1)	0.037 (2)	0.038 (1)
*U*_eq_(*Y*4)	0.083 (3)	0.082 (3)	0.044 (2)
*U*_eq_(*Y*5)	0.112 (5)	0.116 (5)	0.061 (3)
ΔMSDA(*X*4—*Y*4)	0.052 (8)	0.036 (8)	0.010 (6)
ΔMSDA(*X*5—*Y*5)	0.040 (11)	0.041 (11)	0.008 (7)

Note: (*a*) data taken from Zuhayra *et al.* (2008 ▸).
